# A Patient with Berardinelli-Seip Syndrome, Novel *AGPAT2* Splicesite Mutation and Concomitant Development of Non-diabetic Polyneuropathy

**DOI:** 10.4274/jcrpe.galenos.2018.2018.0227

**Published:** 2019-09-03

**Authors:** Joanna Oswiecimska, Mateusz Dawidziuk, Tomasz Gambin, Katarzyna Ziora, Marta Marek, Sylwia Rzonca, D. Lys Guilbride, Shalini N. Jhangiani, Anna Obuchowicz, Alicja Sikora, James R. Lupski, Wojciech Wiszniewski, Pawel Gawlinski

**Affiliations:** 1 Medical University of Silesia in Katowice, Department of Pediatrics in Zabrze, Silesia, Poland; 2 Institute of Mother and Child, Department of Medical Genetics, Warsaw, Poland; 3 Warsaw University of Technology, Institute of Computer Science, Warsaw, Poland; 4 Baylor College of Medicine, Department of Molecular and Human Genetics, Texas, USA; 5 Medical University of Silesia in Katowice, Department of Pediatrics in Bytom, Silesia, Poland; 6 No current affiliation; 7 Human Genome Sequencing Center, Baylor College of Medicine, Texas, USA; 8 Baylor College of Medicine, Department of Pediatrics, Texas, USA; 9 Texas Children’s Hospital, Texas, USA; 10 Oregon Health and Science University, Department of Molecular and Medical Genetics, Portland, USA

**Keywords:** Berardinelli-Seip syndrome, seipinopathy, congenital generalized lipodystrophy, polyneuropathy, AGPAT2, fat biology

## Abstract

Primary polyneuropathy in the context of Seip-Berardinelli type 1 seipinopathy, or congenital generalized lipodystrophy type 1 (CGL1) has not been previously reported. We report the case history of a 27 year old female CGL1 patient presenting with an unusual additional development of non-diabetic peripheral neuropathy and learning disabilities in early adolescence. Whole exome sequencing (WES) of the patient genome identified a novel variant, homozygous for a 52 bp intronic deletion in the *AGPAT2* locus, coding for 1-acylglycerol-3-phosphate O-acyltransferase 2, which is uniquely associated with CGL1 seipinopathies, with no molecular evidence for dual diagnosis. Functional studies using RNA isolated from patient peripheral blood leucocytes showed abnormal RNA splicing resulting in the loss of 25 amino acids from the patient AGPAT2 protein coding sequence. Stability and transcription levels for the misspliced *AGPAT2* mRNA in our patient nonetheless remained normal. Any AGPAT2 protein produced in our patient is therefore likely to be dysfunctional. However, formal linkage of this deletion to the neuropathy observed remains to be shown. The classical clinical presentation of a patient with *AGPAT2*-associated lipodystrophy shows normal cognition and no development of polyneuropathy. Cognitive disabilities and polyneuropathy are features associated exclusively with clinical CGL type 2 arising from seipin (*BSCL2*) gene mutations. This case study suggests that in some genetic contexts, *AGPAT2* mutations can also produce phenotypes with primary polyneuropathy.

**What is already known on this topic?**

Within Berardinelli-Seip syndrome (congenital lipodystrophy disorders characterized by total absence of both metabolic and mechanical fat tissue) patients, only congenital generalized lipodystrophy type 1 (CGL1) retains mechanical fat and is exclusively associated with 1-acylglycerol-3-phosphate O-acyltransferase 2 (*AGPAT2*) gene mutations. Polyneuropathies and learning deficiencies are currently unknown in the context of classical CGL1 disease and *AGPAT2* lesions.

**What this study adds?**

The case history of a patient with classical CGL1 followed for 27 years is presented. The patient, in addition to mechanical fat retention, developed polyneuropathy and learning deficiencies. A new *AGPAT2* intronic deletion was detected in this patient. Our results describe a phenotype expansion for CGL1 and suggest that certain *AGPAT2* gene lesions cause neuropathy which blurs clinical presentation boundaries for CGL and other fat biology disorders.

## Introduction

Berardinelli-Seip syndrome, also known as congenital generalized lipodystrophy (CGL), occurs in approximately 1 in 10 million of the world population and can result from mutation in four genes, giving rise to four clinically similar but distinguishable subsyndromes affecting fat biology (1). CGL type 1 (OMIM#608594) is autosomal, recessive and uniquely associated with mutation in the *AGPAT2* gene encoding 1-acylglycerol-3-phosphate O-acyltransferase 2 (2). This enzyme is integral to phospholipid biosynthesis, triglygeride/fat formation and storage, adipocyte formation and fat metabolism pathways and has multiple molecular interaction partners (1).

Clinical symptoms associated with CGL1 described to date involve both metabolic malfunctions and physical malformations present in all forms of CGL. Complete lack of all metabolic body fat (adipose tissue that stores energy) from birth is the central clinical characteristic for all forms. CGL1 patients alone, however, retain mechanical fat (adipose tissue that provides protective padding for joints and points of impact, i.e. palms, soles of feet, joints, scalp facial bones). This fat distribution is specific and differentially diagnostic for CGL1 (1). Clinical neuropathy and cognitive deficits are associated with CGL2 and mutations in seipin *(BSCL2)*, and are rare but not unknown for CGL3 and CGL4 syndromes [associated with caveolin-1 *(CAV1)* and cavin (RNA polymerase 1 and transcript release factor: *PTRF*) gene mutations, respectively] (1,2). Primary neuropathy and cognitive deficit in the context of CGL1, in the absence of diabetic or other secondary disease complications, are previously unreported traits.

We present the natural history of a female CGL1 patient, continuously recorded from infancy to adulthood. We further demonstrate that this patient carries a previously unknown homozygous intronic deletion variant g.12562_12613del p.(Val197Glufs*32) in the *AGPAT2* gene. Our functional studies show that the deletion disrupts normal *AGPAT2* transcriptional processing and mRNA coding content consistent with a dysfunctional and therefore potentially pathogenic effect for this deletion.

## Case Report

Table 1 lists the clinical symptoms from infancy (three months) to current age (27 years) in chronological order of emergence in this female patient.

Family history disclosed distant parental consanguinity; identity by descent is corroborated by extensive absence of heterozygosity (AOH) totalling 46 Mbp, in the patient genome, with an average AOH region size of 321 Kbp. The AOH block encompassing the patient *AGPAT2* gene is 1.1 Mbp (see Methods). Direct ancestors on both sides lived in the same village for many generations. Both parents are clinically asymptomatic for CGL1. One grandfather however, presented with lipodystrophy and diabetes mellitus (DM) (Column 4, Table 1).

The patient was born to a 34 year old multigravida mother by spontaneous vaginal delivery. The father was 39 years old. Parents reported an unremarkable prenatal history. Clinical evaluation of the patient at age three months revealed the presence of numerous dysmorphic and metabolic features associated with CGL (Table 1) including seven clinical features diagnostic for CGL (1-7, Table 1). Physical examination revealed generalized lipodystophy, large hands and feet, and enlarged tongue (8-10, Table 1), a low anterior hairline and low set ears (11-12, Table 1), hepatomegaly and an umbilical hernia (13, Table 1).

Imaging techniques revealed that the patient had cardiovascular system abnormalities including concentric hypertrophic cardiomyopathy, left ventricle enlargement and thickened intraventricular septum (5, Table 1). Heart muscle contractility was good. Abdominal ultrasound imaging showed a hyperechogenic liver (3, Table 1). Pathological evaluation of a liver biopsy specimen showed microvesicular steatosis and intertrabecular fibrosis (3, Table 1).

Metabolic abnormalities present from birth included elevated serum triglycerides (4.01 mmol/L; normal range 0.4-1.8 mmol/L) and low high-density lipoprotein-cholesterol (HDL-C) (0.58 mmol/L; normal range 0.9-2.0 mmol/L).

Oral glucose tolerance test (OGTT) was normal; both fasting glucose and 120 minute glucose were 4.7 mmol/L and glycated hemoglobin (HbA1c) was 4.82% (normal range 4.5-6.5%). Other abnormal laboratory studies included mildly elevated serum alanine aminotransferase (50.2 U/L; normal range 0-41 U/L) and high alkaline phosphatase (ALP) (351 U/L; normal range 20-150 U/L). All of these metabolic imbalance findings, with the exception of elevated ALP, were progressive conditions (1-6, Table 1).

At age seven years the patient displayed acanthosis nigricans on the nape of the neck, in the axillary and popliteal regions and had prominent musculature, due to general absence of metabolic fat tissue and abnormal fat deposition in muscles, (14-17, Table 1). At this age it was noted that despite absence of metabolic fat, mechanical fat tissue was maintained. Accelerated linear growth velocity was evident, concomitant with an accelerated skeletal maturation of two years. Serum growth hormone concentration was low [0.83 ng/mL (result below 1 ng/mL excludes acromegaly)] and a magnetic resonance imaging (MRI) scan of the hypophysis was normal. Gigantism was not observed, despite the appearance of acromegaloid features.

At age 14, neurological symptoms (18-22, Table 1) began to emerge. Learning disability (IQ score 88) (18, Table 1) was first noted at this age. We also noted acroparaesthaesiae in both hands (19, Table 1). In addition we found changes in electromyogram (EMG) tracings, a mildly reduced neurogenic pattern and decreased motor fibre nerve conduction velocity (NCV) in median, sural and peroneal nerves which was also present in the sensory fibres of the median and sural nerves (20-22, Table 1). MRI scans however showed no sign of median nerve compression. Normal serum calcium, phosphorus, magnesium and parathyroid hormone concentrations further excluded hypoparathyroidism.

At age 16, the patient presented with multiple endocrine abnormalities. At this time a number of clinical features emerged relating to hormonal disturbances (23-30, 32 Table 1). She presented with primary amenorrhoea with clinical and laboratory findings of hyperandrogenism including clitoral enlargement, elevated free testosterone 11.33 pg/mL (normal range 1.1-6.3 pg/mL) and elevated androstenedione 3.60 ng/mL (normal range 0.8-2.4 ng/mL) (28,30 Table 1). Sonographic evaluation showed atrophic ovaries with no ovarian follicles (28, Table 1). Hirsutism was also first noted at this age (32, Table 1). Hormonal function of the hypophysis was normal.

The patient also developed DM (27, Table 1) identified on the basis of an OGTT (0 min - 3.7 mmol/L; 120 min - 11.9 mmol/L) with a high homeostasis model assessment of insulin resistance value [10.39 (normal <2.5)]. HbA1c was 4.6%.

**Current status: **At 27 years, our patient has graduated from college and is employed as a clerk in an office. Paraesthesiae of the hands has not worsened since its first appearance. Diabetes is well-controlled (HbA1c - 5.1%), but dyslipidemia persists despite aggressive therapy (serum triglycerides - 3.8 mmol/L; HDL-C - 0.29 mmol/L). Current medications include metformin (3 g/day), fenofibrate (267 mg/day), rosuvastatin (10 mg/day) and insulin (1.5 UI/kg/day).

### Molecular Analyses

WES analysis revealed a homozygous 52 bp intronic deletion, g.12562_12613del p.(Val197Glufs*32), affecting the 5’splice site for exon 5 of the *AGPAT2* gene. Bioinformatic prediction software (MutationTaster) indicates the deletion (g.12562_12613del) is of unknown pathogenicity. This variant is absent from the ExAC and 1000 G databases. No rare variant alleles in other known disease associated genes were found which could potentially explain the lipodystrophy phenotype or the neuropathy observed in our patient (3).

**Figure 1:**
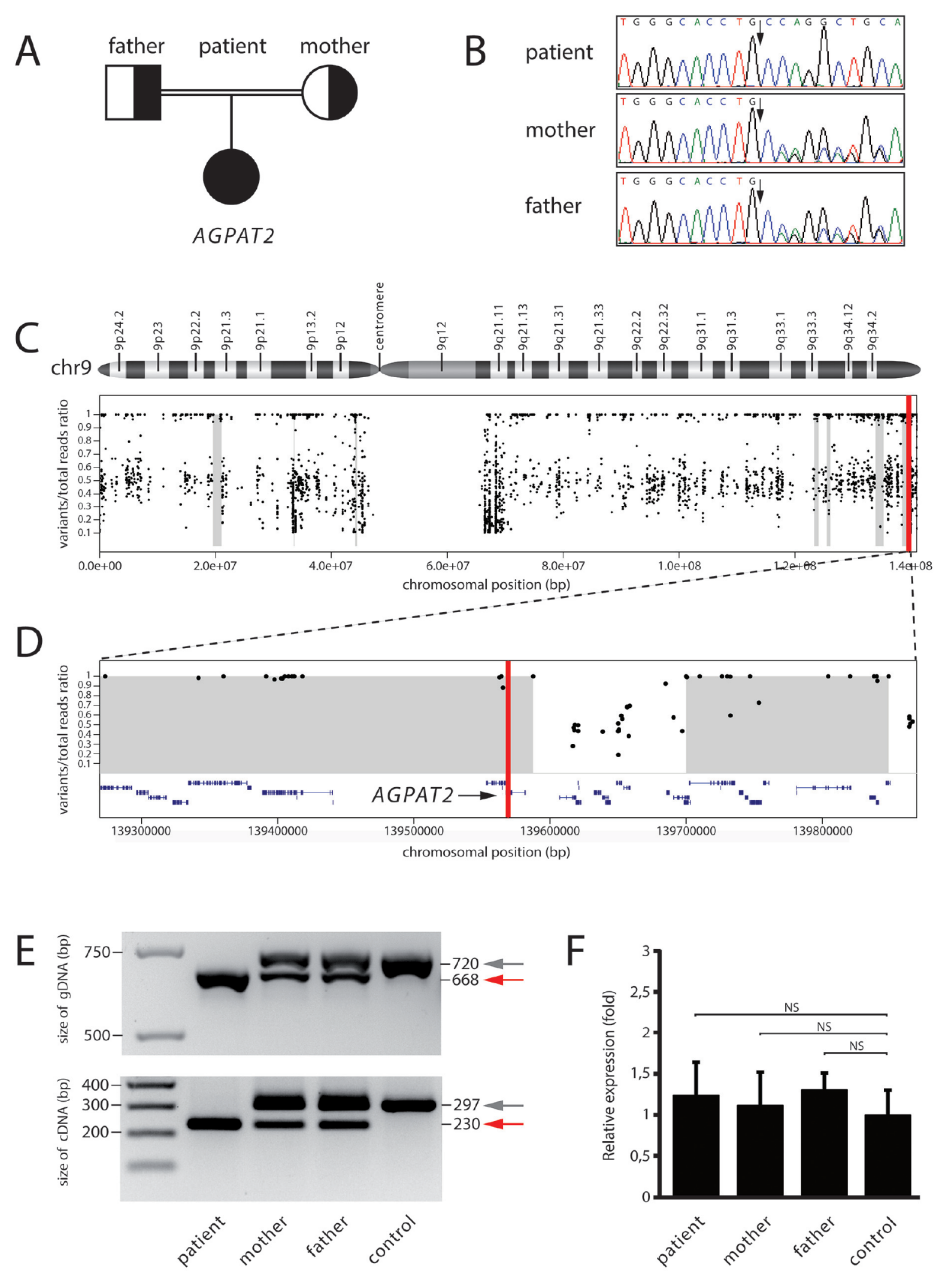
A) Patient pedigree for homozygous *AGPAT2* deletion mutation c.589-55_589-4del p.(Val197Glufs*32). B) Sanger sequence confirmation of biparental inheritance for the mutation. Black arrow shows point of deletion. C) Genomic context of *AGPAT2* mutation. Cartoon shows Chromosome 9 organization. Grey blocks denote regions with absence of heterozygosity (AOH). Scattered dots indicate single nucleotide variation (SNV) for proband along chromosome 9. Absence of SNV in centromere-adjacent areas reflects lack of reference sequence for these region. D) Detail for AOH region surrounding the *AGPAT2* mutation (vertical red line at around 13 958 000 base pair). E) PCR products for *AGPAT2* in patient (mutation homozygous), each parent (mutation heterozygous) and control individual (wildtype homozygous). Red arrowhead, reduced *AGPAT2* product size reflecting deletion mutation. Grey arrowhead, normal size wildtype*AGPAT2* product. Genomic gDNA template, upper image; complementary cDNA template lower image. F) Real time-polymerase chain reaction products for *AGPAT2* mRNA expression levels in patient, parents and control are comparable NS: non-significant

Sanger sequencing confirmed the *AGPAT2* deletion variant and cosegregation with the disease trait according to Mendelian expectations (Figure 1A) and also showed the expected reference sequence around the deletion at the nucleotide level (Figure 1B). This shows the patient is homozygous for the g.12562_12613del p.(Val197Glufs*32) allele. Also, each clinically asymptomatic parent is heterozygous for the identical variant allele (Figure 1C, 1D).

Standard polymerase chain reaction (PCR) (PCR; see Figure 1E, upper image) on genomic template DNA from both patient and parents generated a PCR product shorter (red arrowhead) than the wildtype (grey arrowhead), consistent with a 52 bp genomic deletion. Standard PCR on cDNA templates (lower image) revealed a PCR product shorter by 75 bp for the patient *AGPAT2* mRNA (red arrow) relative to the wildtype control individual mRNA (grey arrowhead). Direct Sanger sequence of the cDNA PCR products showed complete deletion of exon 5 (75 bp), leaving exon 4 joined to exon 6 with a frameshifted coding sequence downstream of the join, creating a premature translation stop signal. The parents are each heterozygous for the deletion and generate both forms of mRNA, and therefore show both mutated and wildtype PCR products (Figure 1E, mother, father).

To assess mutant *AGPAT2* mRNA expression levels and/or stability we used real time-PCR (RT-PCR) to quantify mutant and WT *AGPAT2* mRNA. Expression levels of *AGPAT2* mRNA generated from patient (homozygous for deletion allele) and both parent (heterozygous for deletion allelle) mRNA samples are comparable to those of a healthy control individual (homozygous for wiltdtype allele) (Figure 1F). Stability and/or transcription levels therefore appear unaffected for the mutant mRNA. We concluded that *AGPAT2* mRNA expression levels and stability in our patient remain unaffected by this deletion.

### Methods Used in the Genetic Analysis

Genomic DNA samples were isolated from blood leucocytes from each individual using automatic magnetic bead-based method (MagnaPure, Roche). Copy number variations were identified using array Comparative Genomic Hybridization (aCGH: CytoSure Constitutional v3 8x60K, Oxford Gene Technology) and bioinformatic analyses using XHMM (4) and HMZDelFinder (5) algorithms; single nucleotide variation was determined by WES analysis (6), and confirmed by Sanger sequencing. Chromosomal regions demonstrating AOH were detected by analyzing B-allele frequency data obtained from WES (6) by running BafCalculator accessible from https://github.com/BCM-Lupskilab/BafCalculator (7). *AGPAT2* expression was measured by quantitative RT-PCR (TaqMan Gene Expression Assay for *AGPAT2* gene, Life Technologies, Grand Island, NY, USA), on blood lymphocyte mRNA isolated using High-Capacity cDNA Reverse Transcription Kit (Life Technologies, Grand Island, NY, USA). Level of *AGPAT2* expression was corrected to the mRNA level of the housekeeping genes GAPDH and TBN. Expression data reflected the means of three independent experiments each performed in triplicate.

**Primers and probes:**

gDNA PCR

F: CTCACTGGCTTCCTGAGATGG; R: GGTCCATCCGTGTGAAGTCT

cDNA PCR

F: GGGAGAACCTCAAAGTGTGG; R: GGTCTTGGAGATGTGGAGGA

RT-PCR

TaqMan Gene Expression Assay, Thermofisher labelled probes cat. no. HS00944961.

The study was approved by the Bioethics Committee of the Institute of Mother and Child, Warsaw. Informed consent was obtained from the patient and her parents.

## Discussion

The detailed, lifelong clinical case history revealed findings diagnostic for Berardinelli-Seip syndrome from infancy. Childhood mechanical fat distribution was diagnostic for CGL1. This patient also showed development of polyneuropathy and cognitive disability in early adolescence, symptoms not previously reported in CGL1 patients.

Intellectual disability is typical of CGL2 and rare in other forms of CGL. Primary neuropathy in the absence of DM complications leading to neural pathology, has been associated with CGL2 but has not been reported for CGL1. Polyneuropathy has been associated with a range of lipodystrophic disorders, but in CGL1 patients, the neuropathy reported to date arises from diabetic complications or other secondary conditions. In our patient, laboratory evidence of diabetes was only found two years after initial development of neuropathy. We suggest that the polyneuropathy observed was therefore unlikely to be a diabetic complication. EMG/NCV results (see 20-21, Table 1) suggest demyelinating neuropathy, similar to that caused by duplications in the *PMP22* gene, responsible for Charcot-Marie Tooth (CMT) type 1A syndrome. However, no *PMP22* duplication was detected, nor were any recessive CMT genes found to map within AOH intervals. The possibility that elevated patient triglyceride levels contribute to the clinical manifestation of peripheral neuropathy however, cannot be excluded.

All forms of CGL involve complete lack of metabolic fat (body fat) from birth and the majority show early development of severe hypertriglyceridemia, hepatic steatosis, hepatosplenomegaly, acanthosis nigricans and insulin resistance, generally leading to diabetes in early adolescence. Enlargement of liver tissue and slightly enlarged hands and feet are also typical. Myocardiopathies arise in approximately 25% of individuals. In the case described here the emergence at age 16 of multiple symptoms related to hormonal disturbances after puberty (eg. polycystic ovarian syndrome and hyperinsulinemia) is also typical for all Berardinelli-Seip syndromes including CGL1. Our patient presented with all these CGL-associated symptoms by early-mid adolescence, with concomitant emergence of neuropathological symptoms and learning disability in early adolescence (8). In addition, umbilical hernia, present in our patient at three months, was reported to be associated only with *BSCL2* mutations in one patient (9). The clinical picture therefore suggests CGL2, despite the normal mechanical fat distribution differentially diagnostic for CGL1 (1).

Our genomic investigation nonetheless confirms a previously unknown, single exon, homozygous 52bp deletion in *AGPAT2*; a gene uniquely associated with CGL1 seipinopathy. Given the inability to identify any other known disease genes that might explain the unusual phenotypic features (i.e. polyneuropathy, cognitive deficiency) associated with known *bona fide* *AGPAT2*-related CGL1 clinical manifestations in this patient, we suggest a potential phenotypic expansion. Whether this new mutation causes the neuropathology observed however, remains unresolved. Our molecular analyses show the intronic 5’splice site deletion eliminates 25 codons of protein coding sequence and generates a frameshift resulting in a premature translation stop codon. There is no evidence for mutant *AGPAT2* mRNA instability in the blood cell studies. Structure and function of any AGPAT2 protein in our patient however would likely be impaired.

Precisely how this would affect physiological pathways involving *AGPAT2* is unknown. The AGPAT2 protein is located in the membrane of the endoplasmic reticulum and is primarily involved in triglyceride and phospholipid biosynthesis, with multiple interaction partners involved in lipid biosynthesis/degradation and related pathways. These include acyl chain remodelling of phosphatidylethanolamine (10), adipose droplet formation, lipid signalling and ER and mitochondrial membrane transport pathways (11,12). Many CMT neuropathy genes involve this transport biology, hinting at possible overlapping molecular bases for the polyneuropathy observed in this patient. Disruption of post-translational protein-protein interactions central to lipid homeostasis and of related pathway function, such as cholesterol metabolism, are highly probable and likely to have fundamental physiological effects. Other mutations leading to similar disequilibrium of lipid homeostasis, phospholipid degradation and remodelling in ER and mitochondrial membranes for example, have been linked to neural degeneration and epileptic seizures in other species including flies and worms, with similar phenotypes for mutations in human gene counterparts (13). Disruption of phospholipid homeostasis has been reported to be associated with α-synuclein protein aggregation, implicated in the pathology of Parkinson’s disease (11,14). Disrupted cholesterol metabolism has also been linked to protein aggregation leading to mitochondrial distribution defects and neurodegenerative disease (15,16).

It is notable that known *AGPAT2* regulatory circular RNA (circRNA) (circRNAs; non-coding post-transcriptional splicing products) expression levels are high in normal foetal tissues, including adrenal tissue which regulates circulating hormonal levels in the developing foetus (17,18). Recent elucidation of fundamental functions for circRNA in eukaryotic gene expression programs (19) has highlighted the potential for future investigations into defective RNA processing during foetal development as a possible contributor to genetic disorders.

Finally, we would add an epidemiological note. *AGPAT2* mutations predominate in American CGL cases of African descent. The vast majority of European seipinopathies arise from mutations in *BSCL2* (20). The newly identified *AGPAT2* deletion in our patient was located within a large block (approximately 1.1 Mb) of a chromosome sequence with both alleles identical at nucleotide sequence level (Figure 1C, 1D). Blocks of AOH arise when parentage is related, as was the case for this patient. The rare deletion in *AGPAT2* identified appears to have arisen in an individual from a small European village and accumulated in the relatively static local population over generations.

This case is the first report of primary polyneuropathy within the classical clinical CGL1 syndrome exhibiting differentially diagnostic mechanical fat retention, establishing a potential phenotypic expansion for CGL1 disease. We further identified a new, recessive intronic splice site deletion in the CGL1-associated *AGPAT2* locus, resulting in an apparently translatable truncated mRNA species with missense coding. Precisely how the splicing defect identified affects AGPAT2 protein physiology or noncoding transcriptional regulatory RNA functions remains undefined. This however, is the case for all *AGPAT2* mutations linked to a CGL1 phenotype and the mechanism of action has not been defined for any thus far (12).

## Figures and Tables

**Table 1:**
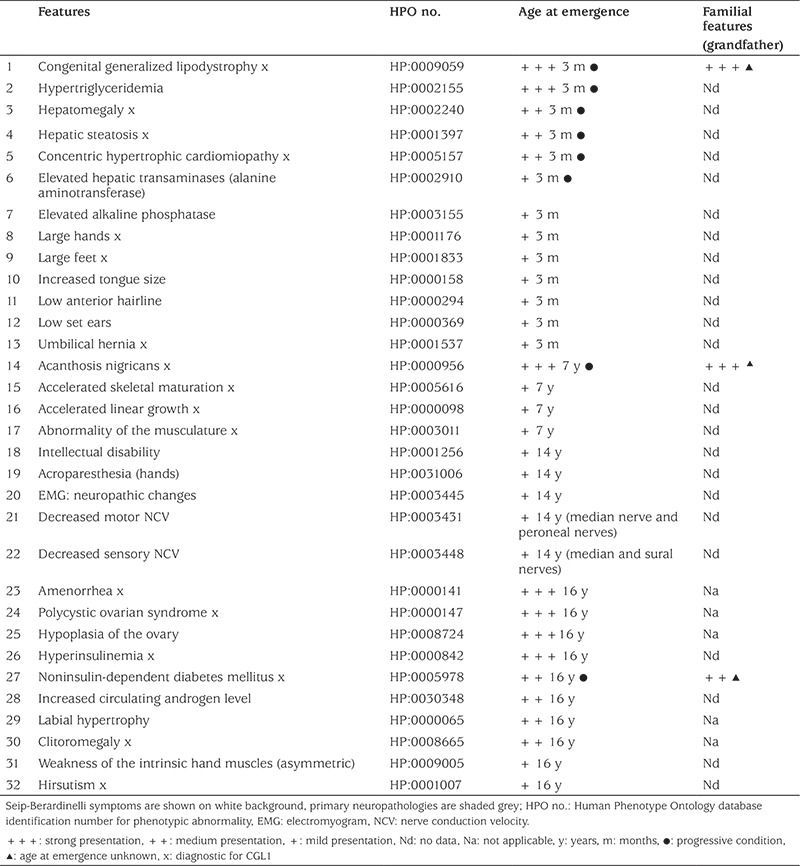
Developmental timeline for clinical emergence of Seip-Berardinelli syndrome and neuropathology features in our patient
